# Novel methodology for determining the effect of adsorbates on human enamel acid dissolution

**DOI:** 10.1016/j.archoralbio.2017.09.035

**Published:** 2018-01

**Authors:** N. Pechlivani, D.A. Devine, P.D. Marsh, A. Mighell, S.J. Brookes

**Affiliations:** Department of Oral Biology, School of Dentistry, Wellcome Trust Brenner Building, St. James’s University Hospital, University of Leeds, Leeds, LS9 7TF, UK

**Keywords:** Adsorbates, Acid demineralisation, Demineralisation inhibitors, Salivary protein, Human enamel

## Abstract

**Objective:**

The effect of various interventions on enamel demineralisation can be determined by chemically measuring mineral ions dissolved by the attacking acid. Results are usually expressed as mineral loss per surface area of enamel exposed. Acid resistant varnish or adhesive tape are typically used to delineate an area of enamel. However, enamel surface curvature, rugosity and porosity reduce the reliability of simple area measurements made at the macro scale. Our aim was to develop a simple method for investigating the effect of adsorbates on enamel demineralisation that does not rely on knowing the area of enamel exposed. As an exemplar we have used salivary proteins as a model adsorbate.

**Design:**

Natural human tooth enamel surfaces were subjected to five sequential acid challenges and then incubated in adsorbate (whole clarified saliva) followed by a further 15 acid challenges. Demineralisation was determined by measuring the phosphate released into the acid during each exposure by a spectrophotometric assay. The initial five challenges established a mean baseline mineral loss for each tooth against which the effect of subsequently adsorbed proteins could be compared.

**Results:**

Salivary proteins significantly reduced the acid demineralisation of human enamel by 43% (p < 0.01). Loss of proteins during each challenge corresponded to a gradual reduction in the degree of protection afforded.

**Conclusions:**

The methodology provides a simple and flexible means to investigate the effect of any adsorbate on enamel acid dissolution. Knowledge of the area of exposed enamel is irrelevant as each tooth acts as its own negative control.

## Highlights

• Method for investigating effect of adsorbates on acid dissolution of enamel.
• Effect of repeated acid exposures on adsorbates can be measured over time.
• Specific salivary proteins significantly reduced acid demineralisation of enamel.
• Desorption of specific proteins corresponds to reduction in protection against acid.

## 1 Introduction

The effect of adsorbates on hydroxyapatite surfaces is of clinical interest as they may influence demineralisation. Adsorbates of interest include salivary proteins that are selectively adsorbed by enamel surfaces to form the pellicle layer which can reduce acid dissolution of enamel (Hannig et al., 2004) and other non-salivary peptides species such as those derived from casein phosphopeptides (Reynolds, Riley, & Storey, 1982), and dentin phosphoprotein derivatives (Yang et al., 2016).

The most common methods of determining the protective effects of adsorbates against acid demineralisation of enamel include microradiography (to quantify lesion depth and volume) (Hall et al., 1999), scanning electron microscopy (to visualise erosive alterations of the enamel surface) (Hannig et al., 2004), transmission electron microscopy (to assess the resistance of the pellicle to acid attack) (Hannig & Balz, 2001), measurement of surface microhardness (Bruvo, Moe, Kirkeby, Vorum, & Bardow, 2009) and direct determination of calcium and phosphate ions released from the enamel into the attacking acid (Nekrashevych & Stosser, 2003). The direct measurement of calcium and phosphate ions released from enamel, either by chemical colorimetric assays (Attin, Becker, Hannig, Buchalla, & Hilgers, 2005; Brookes, Shore, Robinson, Wood, & Kirkham, 2003) or atomic absorption spectroscopy (Hannig et al., 2004; Hannig, Hess, Hoth-Hannig, & De Vrese, 2003; Nekrashevych & Stosser, 2003; Siqueira, Margolis, Helmerhorst, Mendes, & Oppenheim, 2010; Wiegand, Bliggenstorfer, Magalhaes, Sener, & Attin, 2008), is arguably the most sensitive means of quantifying mineral loss. With these direct methods, a typical experimental approach is to quantify the mineral ions lost (i.e. mineral dissolved) per surface area of exposed enamel. Known areas of enamel are delineated by painting tooth crowns with nail varnish to leave an exposed window whose area can be measured (Hannig et al., 2003; Featherstone, Behrman, & Bell, 1993; Siqueira et al., 2010;). However, making such measurements on the macro scale can be difficult due to the curvature of the enamel surface. In addition, micro features associated with the surface (e.g. varying degrees of rugosity and sub-surface porosities accessible to acid) may mean that a simple gross measurement of window area under-estimates the true area of the enamel exposed.

Although guidelines for good methodologies for use in initial erosion models have been published (Young & Tenuta, 2011), there is a need for standardisation of the conditions used for studying the acid demineralisation of human enamel with relation to protein adsorbates. In this paper we report a simple, reliable and inexpensive method for determining the protective effect of adsorbates (using salivary proteins as a model exemplar) against acid demineralisation of natural enamel surfaces. Unlike other methods, the method does not rely on knowing the surface area of enamel exposed and separate negative control samples (controls without adsorbates) are not required as each sample acts as its own negative control.

## 2 Materials and methods

### 2.1 Volunteers and collection of whole saliva

Ethical approval from the Dental Research Ethics Committee (DREC No: 090212/SB/80) was obtained in order to carry out the experiments using saliva from healthy volunteers not complaining of dry mouth. A signed consent form was obtained from the volunteers after they had read the participant information sheet.

Volunteers (n = 5, 2 males and 3 females, aged 26–50 y) were asked to refrain from eating, drinking or smoking for one hour prior to the saliva collection, which occurred between 9 and 11 am on the day of the experiment. Volunteers were asked to chew paraffin wax (Parafilm) and the stimulated whole saliva was collected by drooling into 1.5 mL micro-centrifuge tubes. The saliva was immediately clarified by centrifugation at 20800*g* for 10 min to remove bacteria, cells and other debris, and used immediately.

### 2.2 Preparation of the enamel samples

The use of sound permanent human teeth for the study was approved by the Dental Research Ethics Committee of the University of Leeds (DREC No: 070213/NP/92). Human permanent molars and premolars were obtained from the Skeletal Tissues Research Bank of the School of Dentistry at the University of Leeds and stored in 70% (v/v) ethanol. Prior to use, the teeth were thoroughly cleaned using pumice powder and a toothbrush, extensively rinsed in distilled water and then cut in half (mesio-distally) using a precision cutting machine (Accutom-5, Struers, Denmark). The root and all cut surfaces were covered with nail varnish, leaving only the natural enamel surface exposed. The tooth halves were rinsed with distilled water, air dried overnight and then stored in 20% ethanol until required (samples were rinsed in distilled water before use).

### 2.3 Acid demineralisation of enamel surfaces

**Fig. 1:**
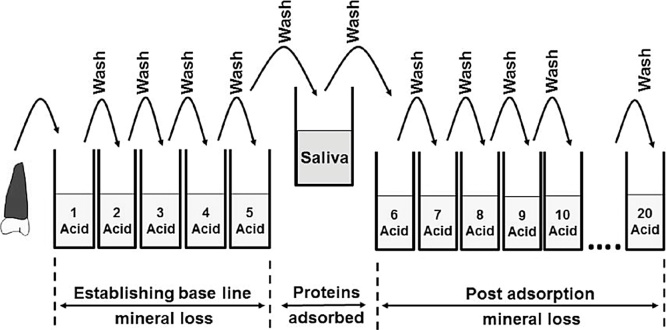
Diagram summarising the experimental methodology.

A tooth half from a single tooth (prepared as described above) was immersed in 1 mL of 10 mM acetic acid (pH 3.3) and gently agitated for 30 s by tapping the tube with a finger. It was then rinsed in distilled water, excess water blotted off with a paper towel and the acid challenge repeated, as described above, using a fresh vial of acetic acid. This process was repeated a further three times giving a total of five sequential acid challenges; the mineral lost in each of the five vials (measured as described in the next section) was used to establish the baseline mineral loss experienced by each tooth half during the acid challenges. This novel approach does away with the need for a separate negative control that is not exposed to the adsorbate in question; the base line mineral loss in the absence of the adsorbate is in effect the negative control. The tooth half was then incubated in 1 mL of whole saliva for 10 min at 37 °C with agitation at ∼150 rpm. It was rinsed with distilled water to remove excess saliva and blotted dry with a paper towel and was subjected to a further 15 sequential acid challenges as described above. Although the method does not require a separate negative control for validation purposes the remaining half of the tooth was treated in the same way except, instead of being agitated in whole saliva for 10 min, it was agitated for 10 min in 50 mM Tris (pH 7.4). The procedure is summarised in Fig. 1.

### 2.4 Determination of mineral lost during each acid exposure: measurement of phosphate

The level of the acid demineralisation was determined by measuring the phosphate released into the acid solution during acid attack. Phosphate was measured using a modified version of the spectrophotometric phosphomolybdate assay as published previously (Brookes et al., 2003). Briefly, a 96 well microplate (Greiner Bio-One Ltd, Stonehouse, Great Britain) was used and 100 μL of sample and 100 μL of “Reagent A” were added to each well in duplicate. Reagent A comprised four volumes of 1.5 mol/L sulphuric acid containing 0.625% (w/v) ammonium molybdate solution, to which one volume of 10% (w/v) ascorbic acid was added just before use. Standard phosphate solutions containing 20, 10, 5, 2.5 and 1.25 μg/mL were used to develop a standard curve with distilled water acting as a blank. After two hours incubation at 37 °C, the absorbance of the samples was measured at 820 nm using a microplate reader (Thermo Scientific Varioskan Flash). The samples were quantified by linear regression based on the standard curve using Skanit Software supplied with the plate reader.

In some experiments, we wished to test if mineral ions in the saliva had an artefactual effect on the phosphate assay. The concentration of free ions in saliva was reduced by buffer exchange using 50 mM Tris pH 7.4. Whole saliva (1.5 mL) was concentrated to 200 μL using an Amicon ultra-centrifugal filter device with 4–5000 MW cut off (Merck Millipore, Darmstadt, Germany). The sample was then made back up to 1.5 mL volume with 50 mM Tris pH 7.4. The phosphate concentration of the saliva sample was measured before and after the dialysis to ensure that the dialysis procedure was effective. The aim of this procedure was to investigate any carryover of phosphate associated with the adsorbed salivary proteins that could confound the phosphomolybdate analysis of phosphate released by acid demineralisation of the enamel.

### 2.5 Statistical analysis

Phosphate released by demineralisation into each vial was normalised to the amount of phosphate dissolved in vial one which was always defined as 100%. Data were analysed using Excel (Microsoft) to calculate the means and standard deviations for the phosphate dissolved in each vial. The mean of the phosphate dissolved in the first 5 acid challenges (vials one to five), before saliva treatment, was compared with the phosphate present in the following vials (vials 6–20) and the differences analysed using a paired *t*-test. *P* values less than 0.05 were considered statistically significant.

## 3 Results

**The protective effect of whole salivary proteins against acid demineralisation of human enamel surfaces**

**Fig. 2:**
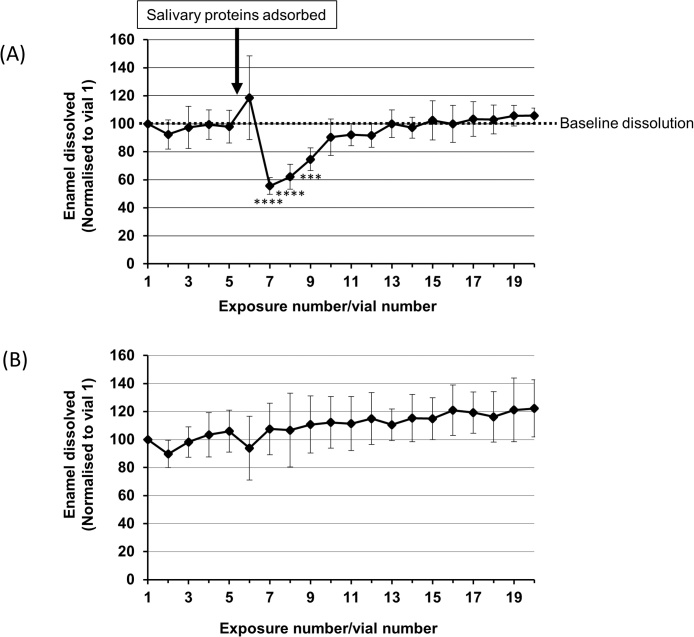
Mineral/phosphate lost from tooth surfaces exposed to acetic acid, pH 3.30, (A) in the presence of saliva and (B) with no saliva. Data in (A) are the means of results using saliva from five volunteers (±SD) with n = 3 for each volunteer (*p < 0.05; **p < 0.01). Data were normalised to the amount of phosphate dissolved in the first acid exposure (mineral dissolved during each subsequent exposure is expressed as a percentage of the mineral dissolved in the first acid exposure). The adsorption of whole saliva significantly reduced enamel dissolution and the proteins remained protective during subsequent acid challenges although the protective effect was gradually lost. In the absence of any adsorbates (B) there is a trend for mineral dissolution to increase with each subsequent acid challenge. This may be due to gradually desorbing any pre-existing residual integument on the enamel and continued erosion would conceivably increase acid access to any micro-porosities present.

Fig. 2a shows the normalised data for phosphate released into each vial of acid. The first five acid challenges established a baseline for the amount of enamel dissolved during each challenge with no significant differences between vials. Following adsorption of the whole salivary proteins (indicated by arrow) there was an apparent increase in acid dissolution during the sixth acid exposure but during the next exposure mineral loss was significantly reduced by 43% (p < 0.01) compared with the mean of mineral lost in the first five acid challenges. Moreover, the proteins remained protective in the subsequent acid exposures, although the effect was gradually lost. The loss of protection corresponded to a gradual desorption of salivary proteins during each acid challenge and, even though protein remained adsorbed after 20 acid challenges, this protein was no longer protective (supplemental Fig. 1). In contrast, control samples (with no salivary protein adsorption) showed no sudden or significant reduction in the mineral released into the vials (Fig. 2b). The mineral lost per acid challenge gradually increases with increasing exposures presumably as the enamel surface becomes more protonated. The apparent decrease in mineral dissolution in control vial 6 following exposure to Tris buffer was not significantly different compared to the mean baseline loss accrued in vial 1–5.

**Fig. 3:**
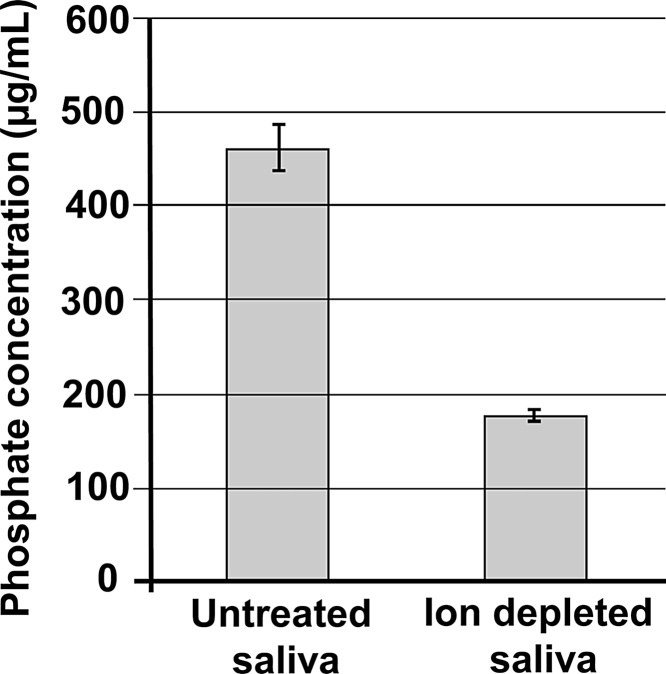
Phosphate concentration in whole saliva before and after dialysis. The phosphate level was significantly (p < 0.05) decreased by 60% in dialysed saliva.

**Fig. 4:**
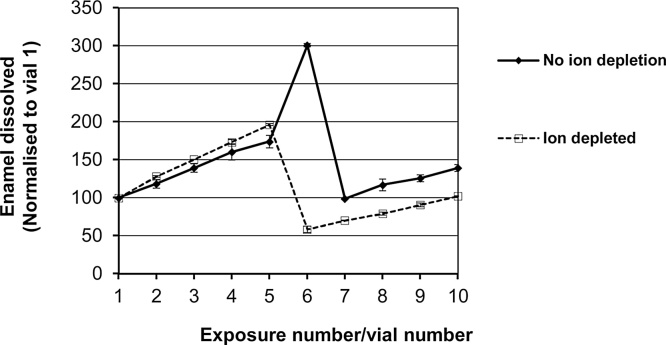
(A) Measurement of phosphate dissolved during each acetic acid (pH 3.30) challenge, before and after exposure to whole saliva and ion depleted saliva. Data were normalised to the amount of phosphate dissolved in the first acid exposure (mineral dissolved during each subsequent exposure is expressed as a percentage of the mineral dissolved in the first acid exposure). The spike of phosphate released during the sixth acid exposure was not apparent when ion depleted saliva was adsorbed to the enamel surface. This suggests inorganic phosphate from saliva loosely associates with the enamel surface or adsorbed proteins giving rise to an artefactual phosphate spike in vial 6.

We hypothesised that the apparent increase in enamel dissolution occurring in vial 6, immediately after the adsorption of salivary proteins, was an artefact caused by the release of labile phosphate ions originating from the saliva that were weakly associated with the newly adsorbed salivary proteins. To test this, we reduced the concentration of ions in the saliva using buffer exchange (ultra-filtration), which reduced the phosphate concentration in the saliva by around two thirds (Fig. 3). Fig. 4 compares the effect of ion depleted saliva to normal whole saliva when serially challenged with acid. There was an obvious spike of phosphate released during the sixth exposure similar to that seen in Fig. 2. In contrast, this spike was absent when ion depleted saliva was adsorbed to the enamel. Note, that these data were acquired using just one enamel surface for the ion depleted saliva and one enamel surface for the non-ion depleted saliva. By chance, both samples lost increasing amounts of mineral during the initial 5 acid exposures; such behaviour is not apparent in Fig. 2a due to the data being derived from mean values.

## 4 Discussion

We have developed a simple, reliable and inexpensive method for determining the protective effect of adsorbed molecules (using salivary proteins as a model exemplar) against acid demineralisation of natural enamel surfaces that overcomes the problems associated with various methodologies used in previous studies. The method employs the sensitive phosphomolybdate assay to measure phosphate dissolved into acid during an acid challenge (though any method for determining the concentration of mineral ions released during acid demineralisation could be used). Acetic acid was used as the demineralising agent. The acid is commonly used in enamel demineralisation studies (Buzalaf, de Moraes Italiani, Kato, Martinhon, & Magalhaes, 2006; Shah, Kosoric, Hector, & Anderson, 2011; Siqueira et al., 2010) and does not affect the phosphomolybdate method used here to determine mineral dissolution. That said, any acid can be used if an appropriate method for determining mineral loss is used (e.g. using atomic adsorption spectroscopy, calcium colorimetry (with Arsenazo III), and ion selective electrodes etc).

Crucially, the method does not rely on knowing the exact area of enamel exposed to achieve meaningful and comparable data. Furthermore, the design allows each enamel adsorbent surface to act as its own negative control (i.e. enamel not treated with an adsorbate) which eliminates any variability due to any biological differences that exist between the experimental and control teeth. Finally, the method also allows adsorbates desorbed from the enamel during a sequential series of acid challenges to be collected and further analysed if required.

In demonstrating a research application for the methodology, we have shown that whole saliva provides significant protection (43%) against acid demineralisation of natural enamel surfaces which is in keeping with many previous reports that adsorbed salivary proteins have the potential to protect enamel from acid dissolution (Carpenter et al., 2014, Delecrode et al., 2015, Hannig and Balz, 2001, Hannig et al., 2003, Nekrashevych and Stosser, 2003; Wetton, Hughes, Newcombe, & Addy, 2007). The method was designed to test the protective effect of saliva after a number of short exposures of 30 s to acetic acid and enabled any proteins desorbed during each exposure to be retained for analysis. Diminished or elevated levels of specific salivary proteins may be associated with susceptibility to dental caries and acid demineralisation of enamel (Guo & Shi, 2013). The benefit of multiple exposures over a single exposure is that it allows the duration of the protective effect against multiple episodes of acid attacks to be investigated. For example, in the current application, repeated acid challenges gradually desorbed some proteins which corresponded to a loss of protection even though some proteins remained adsorbed to the enamel throughout the challenge (supplemental Fig. 1). Previous work has also shown that specific salivary proteins remain adsorbed to enamel following a severe erosive challenge attack (Hannig and Balz, 2001, Hannig et al., 2003) and it has been reasonably suggested that these acid resistant proteins may be protective against acid attack (Delecrode et al., 2015). A methodology based on a single acid challenge would support this view. However, here we are able to show that even though these acid resilient proteins remain adsorbed to the enamel in the face of an acid challenge they provide no protection. Rather, the methodology reported here indicates that the protective proteins are in fact acid labile and are lost on acid attack with a concomitant loss of protection.

The mineral composition of enamel and the tooth surface can vary among individuals or tooth type (Carvalho & Lussi, 2015) and these are important factors to consider in experimental designs. The advantage of the current methodology is that a number of standardised acid challenges are carried out prior to adsorption of the salivary proteins in order to establish the baseline dissolution rate specific to that enamel surface. Following adsorption (of salivary protein in this case), the enamel surface is further challenged with repeated exposures to acid and the effect of the adsorbed substance on dissolution determined. Since each enamel surface acts as its own control, the methodology requires fewer samples compared to situations where separate control and experimental samples are used thereby avoiding a further potential source of variability.

We note that the data presented in Fig. 2a (vial 6), initially look to be at contrary to the conclusion that adsorption of salivary protein reduces enamel dissolution in subsequent acid challenges as there is an apparent increase in the phosphate released during the first acid exposure following protein adsorption. However, when saliva was subjected to ultra-filtration to reduce its ion content (Fig. 3) the apparent phosphate spike in vial 6 was lost (Fig. 4). This suggests that the apparent increase in the phosphate released during the first acid exposure following protein adsorption is an artefact related to phosphate ions associated with adsorbed salivary proteins that are released during the initial acid attack in vial 6. From Fig. 3, it is clear that ultra-filtration could not remove all detectable phosphate ions from the saliva. A similar finding was reported by Baumann et al. (2016) who could not completely deplete saliva of phosphate ions using dialysis presumably, as they suggested, due to phosphate ions binding salivary proteins (Baumann, Kozik, Lussi, & Carvalho, 2016). However, we, and Baumann et al. (2016), analysed phosphate using the phosphomolybdate method in which samples are exposed to concentrated sulphuric acid. Sulphuric acid hydrolysis of inorganic phosphate from salivary phosphoproteins may have also contributed to the inorganic phosphate detected in saliva following either ultra-filtration or dialysis. Certainly, we found that salivary phosphoproteins were released on the first exposure to acid in vial 6 (supplemental Fig. 2). Baumann et al. (2016) also reported that ion-depleted salivary proteins actually provided enhanced protection over non-depleted saliva since phosphate and calcium ions are thought to compete with the proteins for binding sites on the enamel surface. In our study, ion-depleted saliva provided protection against acid attack but we were unable to say if this was superior to the protection provided by non-ion-depleted saliva as the number of replicates used in this ancillary experiment (designed to investigate the apparent increase in phosphate released in vial 6 rather than to quantify the degree of protection afforded by adsorbed proteins) were too low.

The methodology presented here can be easily adapted to carry out more sophisticated studies to model the effect of adsorbed pellicles on enamel dissolution. For example, the acid exposure time and nature of the acid can be changed according to the research question posed. The time allowed for the adsorbates to associate with the enamel surface and the conditions under which adsorption takes place can be varied. Remineralisation steps can be included along with the option to recharge the pellicle to better model the situation in the mouth. As described here, the method involves repeated acid exposures which may modify the physical structure and chemistry of the enamel surface (e.g. protonation) which could influence subsequent adsorption of salivary protein. This models a scenario involving repeated exposure of enamel to an acid beverage (i.e. akin to sipping a drink) followed by a break during which salivary proteins readsorb onto the enamel followed by a further period of repeated exposures to acid (sipping). Inclusion of a remineralisation step, or at least a buffering step, between each acid exposure would negate this potential confounder if required.

Saliva is not the only source of proteins that can adsorb to enamel surfaces and contribute to the pellicle. For example, gingival crevicular fluid contains various proteins including serum proteins and will be present in the immediate environment around the gingival margin. Protective factors such as extraneous ions, dietary proteins, dietary polyphenols, synthetic proteins, and chlorohexidine can inhibit demineralisation directly or by modulating the properties of the adsorbed pellicle (Algarni et al., 2015; Vukosavljevic, Custodio, Buzalaf, Hara, & Siqueira, 2014). Although the method described here uses whole salivary proteins as a model adsorbate, additional applications could include the investigation of the effect of any adsorbed substance; for example, lipids, which were shown to inhibit acid demineralisation of the enamel (Featherstone & Rosenberg, 1984).

In summary, we present a simple methodology for investigating the effect of adsorbates on enamel dissolution and have used it here to illustrate how salivary proteins can be characterised in terms of their ability to protect against enamel dissolution. The methodology is flexible and can easily be developed to model a number of scenarios.
