# Supplementary material for: Novel methodology for determining the effect of adsorbates on human enamel acid dissolution
Source: Arch Oral Biol. 2018 Jan;85:46–50. doi: 10.1016/j.archoralbio.2017.09.035 (PMC5713683; doi:10.1016/j.archoralbio.2017.09.035)
Supplement: Supplementary file 1 [file mmc1.pptx]

## Slide 1
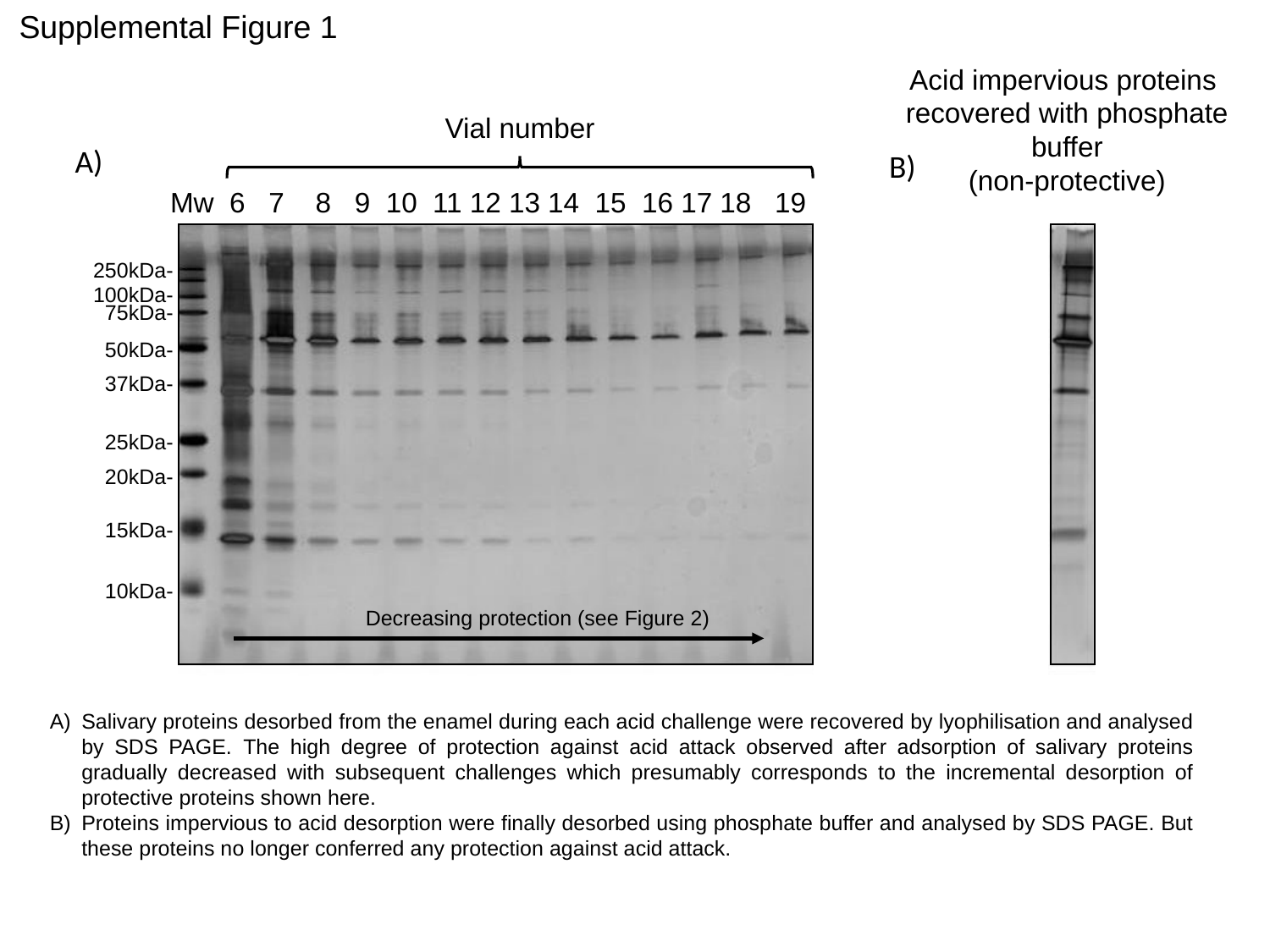

Supplemental Figure 1
Acid impervious proteins
recovered with phosphate buffer
(non-protective)
Vial number
A)
B)
Mw 6 7 8 9 10 11 12 13 14 15 16 17 18 19
250kDa-
100kDa-
75kDa-
50kDa-
37kDa-
25kDa-
20kDa-
15kDa-
10kDa-
Decreasing protection (see Figure 2)
Salivary proteins desorbed from the enamel during each acid challenge were recovered by lyophilisation and analysed by SDS PAGE. The high degree of protection against acid attack observed after adsorption of salivary proteins gradually decreased with subsequent challenges which presumably corresponds to the incremental desorption of protective proteins shown here.
Proteins impervious to acid desorption were finally desorbed using phosphate buffer and analysed by SDS PAGE. But these proteins no longer conferred any protection against acid attack.

## Slide 2
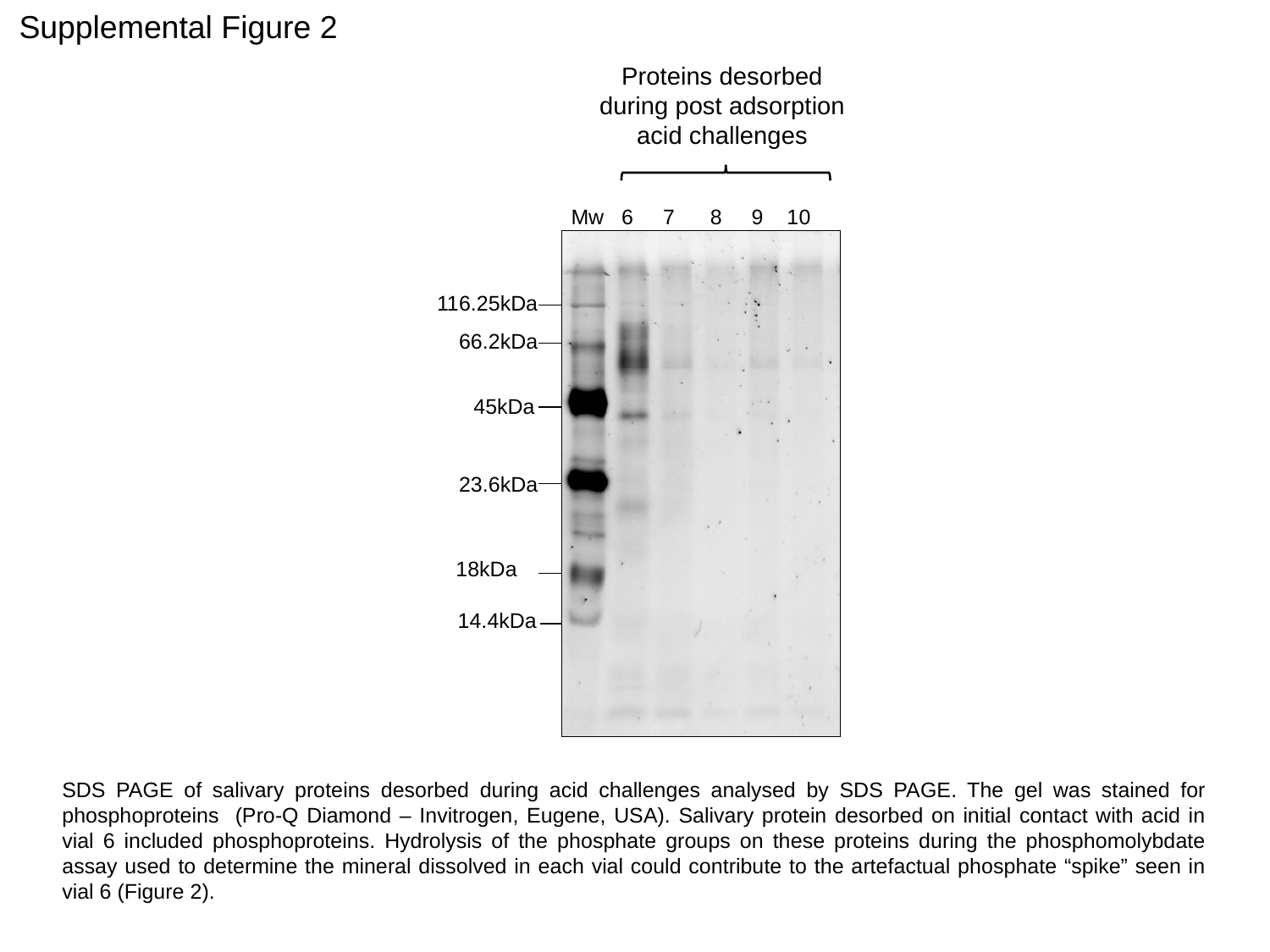

Supplemental Figure 2
Proteins desorbed
during post adsorption
acid challenges
Mw 6 7 8 9 10
45kDa
23.6kDa
116.25kDa
66.2kDa
18kDa
14.4kDa
SDS PAGE of salivary proteins desorbed during acid challenges analysed by SDS PAGE. The gel was stained for phosphoproteins (Pro-Q Diamond – Invitrogen, Eugene, USA). Salivary protein desorbed on initial contact with acid in vial 6 included phosphoproteins. Hydrolysis of the phosphate groups on these proteins during the phosphomolybdate assay used to determine the mineral dissolved in each vial could contribute to the artefactual phosphate “spike” seen in vial 6 (Figure 2).
